# Biomechanical Analysis of Normal Brain Development during the First Year of Life Using Finite Strain Theory

**DOI:** 10.1038/srep37666

**Published:** 2016-12-02

**Authors:** Jeong Chul Kim, Li Wang, Dinggang Shen, Weili Lin

**Affiliations:** 1 Biomedical Research Imaging Center, University of North Carolina at Chapel Hill, Chapel Hill, North Carolina, USA; 2 Department of Diagnostic Radiology, Wake Forest School of Medicine, Winston-Salem, North Carolina, USA; 3 Department of Radiology, University of North Carolina at Chapel Hill, Chapel Hill, North Carolina, USA

## Abstract

The first year of life is the most critical time period for structural and functional development of the human brain. Combining longitudinal MR imaging and finite strain theory, this study aimed to provide new insights into normal brain development through a biomechanical framework. Thirty-three normal infants were longitudinally imaged using MRI from 2 weeks to 1 year of age. Voxel-wise Jacobian determinant was estimated to elucidate volumetric changes while Lagrange strains (both normal and shear strains) were measured to reveal directional growth information every 3 months during the first year of life. Directional normal strain maps revealed that, during the first 6 months, the growth pattern of gray matter is anisotropic and spatially inhomogeneous with higher left-right stretch around the temporal lobe and interhemispheric fissure, anterior-posterior stretch in the frontal and occipital lobes, and superior-inferior stretch in right inferior occipital and right inferior temporal gyri. In contrast, anterior lateral ventricles and insula showed an isotropic stretch pattern. Volumetric and directional growth rates were linearly decreased with age for most of the cortical regions. Our results revealed anisotropic and inhomogeneous brain growth patterns of the human brain during the first year of life using longitudinal MRI and a biomechanical framework.

The human brain exhibits highly dynamic structural and functional changes during early infancy^1^,^2^ as a consequence of interaction between genetic programming, epigenetic and environmental factors. The intracranial brain volume (ICV) increases by 101% in the first year and 15% in the second year, which is about 83% of the adult volume^3^. In the cortex, there is rapid elaboration of new synapses in the first two years of life. Myelination of white matter also proceeds rapidly in the first two years after birth, with the overall pattern of adult myelination completed by the end of the second year of life. However, it is poorly understood how dynamic structural change is related with achievement of psycho-motor and cognitive skills during brain development. In addition, abnormal brain volumetric growths have been widely reported during early infancy and childhood; autism subjects showed brain overgrowth for the first year of life^4^, fragile X syndrome patients exhibited increased caudate and lateral ventricle volumes^5^, and schizophrenic patients showed slower growth rates of white matter^6^. Therefore, this dynamic nature of brain growth requires the sensitive strategies to detect, track and quantify structural change in the brain in spatiotemporal domain^7^.

Using advanced image analysis tools, various parameters related to infant brain development have been evaluated including ICV^3^,^8^, tissue specific volumes, regional brain volumes^9^,^10^, cortical thicknesses, surface areas and gyrification indices^11^. Longitudinal MR studies incorporating automated image segmentation techniques have delineated a rapid and dynamic brain volume growth profile from birth to 2 years of age^3^,^12^,^13^; differential/asymmetric volume growth of different tissue types (gray matter, white matter and cerebrospinal fluid) and functional regions^1^,^8^,^9^ have been reported. In addition tensor-based morphometry (TBM) has also been exploited to identify regional structural differences using the gradients of the nonlinear deformation fields derived from aligning individual images to a common anatomical template^14^,^15^. Using TBM, Jacobian determinant (JD) can be used to discern local volume expansion or contraction relative to the corresponding anatomical structures in a given template^16^. While all of these existing approaches have provided valuable insights into early brain development, none of these approaches provides directionally specific growth information: let us consider an image voxel for which the eigenvalues of the Jacobian matrix are *λ*_1,2,3_ = {1, 2, 0.5}. In such a case the value of JD would be 1 (assuming all off-diagonal components of Jacobian matrix are zeros). Thus, though the directional stretch and shrink exist, this directional change is not able to be detected by only JD^17^,^18^. To this end, the finite strain theory of continuum mechanics was employed as an extension of TBM to offer additional insights into early brain development in this study.

Finite strain theory of continuum mechanics offers an ideal framework to model large deformation phenomena. In particular, its ability to model not only volumetric deformation but also changes of line element’s length and orientation has lent itself to numerous applications on the analysis of elastomers, plastically-deforming materials and biological soft tissues^19^. Leveraging these advantages, Rajagopalan *et al*. utilized finite strain theory to analyze MR images acquired from 38 fetuses. They reported that the most significant changes in the directionality of growth were in the cortical plate located at the major sulci^19^. Furthermore, Studholme and Cardenas showed the orientation of volume changes between abstainers from, and those who relapse to, alcohol use^17^. While these studies have demonstrated the potential advantages of finite strain theory in characterizing brain morphological changes, there are two major limitations. First, deformation of a region of interest (ROI) was decomposed into stretch (scaling) and rotation of infinitesimal elements (polar decomposition). As a result, it is only physically reasonable for one-dimensional case. That is, if a cube object experiences directional stretches and rotations, the deformed cube becomes rectangular cuboid. In reality, biological growth/deformation involves three-dimensional shearing, making the shape of deformed element more complex^20^. Second, a cross-sectional design was employed in the previous studies, making it difficult to exclude potential confounds arisen from inter-subject variability.

To mitigate the above outlined limitations associated with the previous studies and enable characterization of complex three-dimensional brain growth, in this study, Lagrange strain of finite strain theory was employed together with images obtained from a longitudinal imaging study. Lagrange strain tensor models how a volumetric ROI deforms in the coordinate system of reference configuration; diagonal components describe directional stretches and shrinks of line elements whereas off-diagonal components represent rotation of line elements and shearing of a volumetric ROI. Specifically, a volumetric ROI can be modeled as a combination of several line elements. Three-dimensional deformation processes thus can be tracked through each line element and reconstruct a new volumetric ROI in a deformed configuration by applying finite strain theory. In this study, we characterized directionally specific growth behaviors using a cohort of subjects who were longitudinally imaged five times during the first year of life. We aimed to uncover nonlinear, anisotropic and inhomogeneous brain growth and determined age-specific directional and volumetric growth rates during a critical time period of early brain development.

## Results

### Volumetric growth of the brain for the first year of life

**Figure 1:**
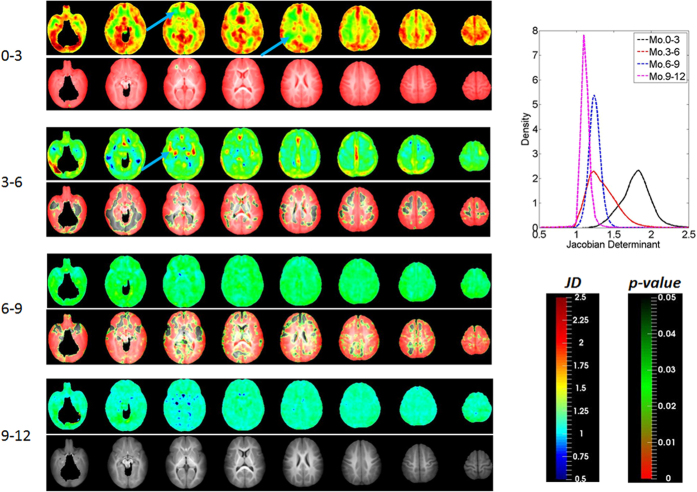
Mean volume growth rates (JD) of the four time periods during the first year of life. The anterior/posterior regions of corona radiate show the lowest local volume change during 0–3 months (arrows). A higher volume expansion at the insula cortex is observed during 3–6 months (arrow). Whole brain distributions of JD during the time periods are shown in the upper right column where x-axis and y-axis represent the JD values and the relative probability density, respectively.

The subjects were scanned at 26 ± 8, 102 ± 25, 189 ± 9, 279 ± 15 and 372 ± 14 postnatal days, respectively. Adjusted mean volumetric growth rates (JD) during the 4 time periods, 0–3, 3–6, 6–9 and 9–12 months, are shown on the Year-1 infant atlas space (Fig. 1). A clear demarcation was observed regarding the characteristics of JD between 0–6 and 6–12 months of age; a substantial volume expansion (JD > 1.0) at the cortical regions is observed during the first 6 months of life, followed by more spatially uniform JD during the second half of the first year. On the other hand, most of the major white matter tracts exhibit a lower JD, suggesting relatively slow growth during the first year of life. The distributions of JD during the four time periods are shown in the upper right column of Fig. 1. Although the distributions of JD are broad during the first 6 months of life, the peak is left-shifted during 3–6 months, suggesting a reduced pace of volume expansion in this time period when compared to that during 0–3 months. Interestingly, although the mean JD is similar for the time periods of 3–6 months and 6–9 months, the distribution is much narrower during 6–9 months. Finally, the mean is further left-shifted and the distribution is much narrower at the time period of 9–12 months when compared to that between 6–9 months.

Mean values of JD and strains of each ROI were computed. Brain regions exhibiting a mean JD greater than 75^th^ percentile (*JD*^0–3^ > 1.91 and *JD*^6–6^ > 1.45) or less than 25^th^ percentile (*JD*^0–3^ < 1.68 and *JD*^3–6^ < 1.20) of all voxels in the brain, including white matter and cerebrospinal fluid, were defined as prominent and slow growing regions, respectively (Tables 1 and 2). Regions with prominent increase of JD are mainly in the right hemisphere adjacent to the interhemispheric fissure (cingulum, lingual, Fusiform, calcarine, cuneus, precuneus, rectus and olfactory gyri). Additionally, some areas of the visual cortex showed higher growth patterns (months 0–12). More importantly, regions related higher cognitive functions showed a higher volumetric growth from month 6 (temporal, parietal, angular and supramarginal gyri). Despite the prominent brain tissue expansion observed in many brain regions, not all brain regions exhibited volume expansion during the first year of life. In particular, some areas of the white matter including anterior corona radiata (months 0–3) and inferior longitudinal fasciculus (months 3–6) showed non-significant volume changes (*p* > 0.05 after FDR correction). Regions in the left hemisphere showed relatively slow volumetric growth during the first 6 months, including cingulum, amygdala, hippocampus, and pallidum. After month 6, temporal pole and frontal pole areas showed lower volumetric growth. Finally, no brain region showed statistically significant volume expansion or contraction in the time period of 9–12 months (*p* > 0.05 after FDR correction) (Fig. 1).

### Direction-specific changes of the brain

**Figure 2:**
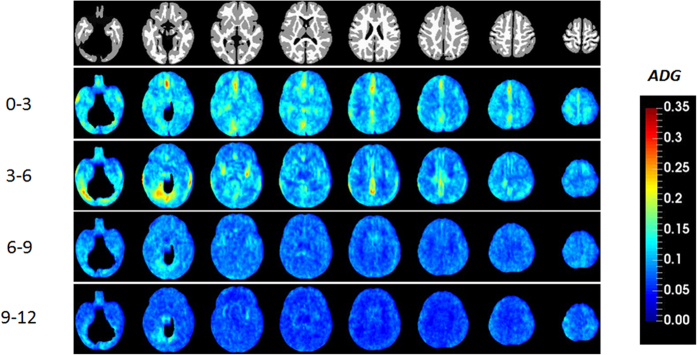
The anisotropy of directional growth (ADG) maps based on directional stretches along the main axes.

**Figure 3:**
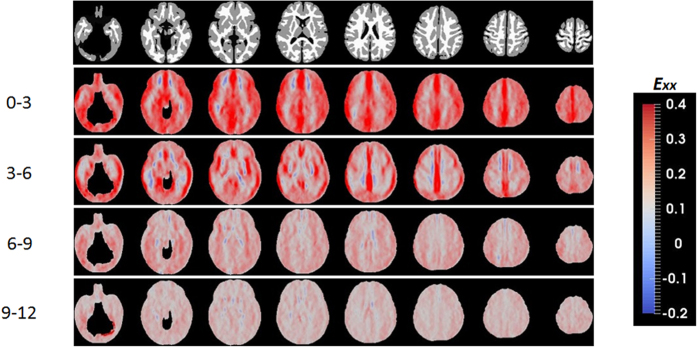
Left to right directional normal strains during the first year of life.

**Figure 4:**
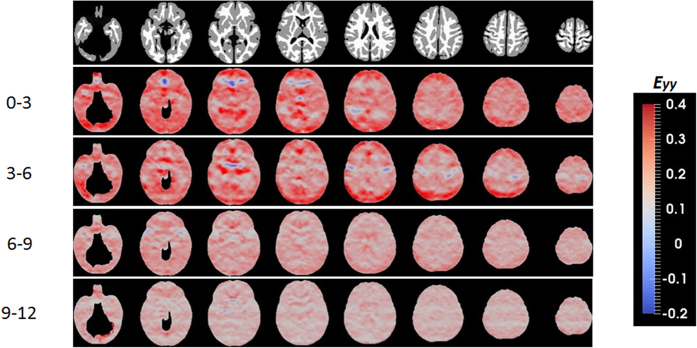
Posterior-anterior directional normal strains during the first year of life.

**Figure 5:**
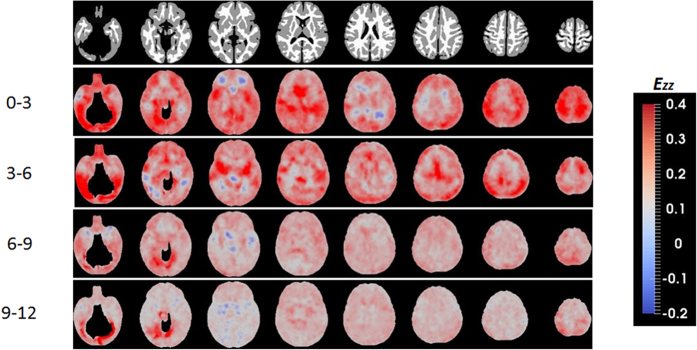
Inferior to superior directional normal strains during the first year of life.

While JD provides insights into early brain development, it does not, however, offer information on directional growth. Figure 2 shows the anisotropy of the directional growth (ADG) during the four time periods whereas the corresponding directional elongations along x (*E*_*xx*_, left-right), y (*E*_*yy*_, anterior-posterior), and z (*E*_*zz*_, superior-inferior) directions are shown in Figs 3, 4 and 5, respectively. Qualitatively, the directional normal strains are spatially non-homogeneous and are age-dependent as shown in ADG maps. These regions showing higher ADG correspond to directionally-specific growth patterns. For the x-direction, remarkably higher rates of changes of normal strains are observed in the interhemispheric fissure and cortical areas for the first 6 months (Table 1). Specifically, right cingulum, right lingual, right olfactory, right recturs, right medial orbitofrontal, and right precuneus gyri showed active x-directional stretches (>75^th^ percentile (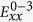 > 0.25 and 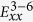 > 0.17) of the whole brain *E*_*xx*_). Even though not statistically significant, slight local contractions in x-direction are observed in the middle cingulums, anterior/posterior corona radiata for the first 6 months. Small x-directional stretches (<25^th^ percentile of the whole brain, (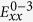 < 0.12 and 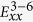 < 0.056)) were mainly at the subcortical areas of the left hemisphere, including amygdala, putatmen, pallidum and caudate (Table 2). For y-directional normal strains, the lateral ventricles, bilateral occipital, bilateral olfactory, bilateral lingual, bilateral cuneus, right superior temporal pole and right angular gyri showed growth rates greater than 75^th^ percentile (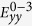 > 0.19 and 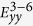 > 0.17) for the first 6 months. Significant local contractions are also observed in y-direction, including the anterior cingulum and anterior regions of corona radiata during the first 3 months (*p* < 0.05 after FDR correction). Left amygdala also showed a low y-directional stretch (<25^th^ percentile of the whole brain during the first three months (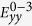 < 0.11 and 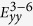 < 0.076)). In z-direction, bilateral fusiform, right inferior occipital, right inferior parietal, left heschl, right inferior temporal gyri and lateral ventricles showed growth rates greater than 75^th^ percentile (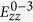 > 0.21 and 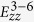 > 0.18) for the first 6 months. After the first 6 months, as observed in JD, more uniform directional stretch patterns were developed, but significant z-directional stretch patterns were observed at bilateral lingual gyri, right fusiform gyrus and right inferior temporal gyrus.

### Shear deformations of the brain

**Figure 6:**
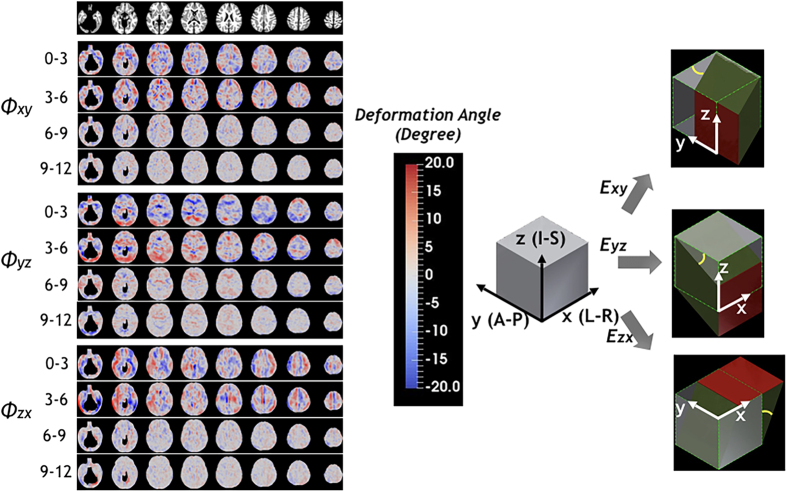
Mean deformation angles exerted by shear strains derived from off-diagonal components of Lagrange strain are showed for every 3 months of the first year of life. Pictorial descriptions of the anticipated deformation profiles with respect to each shear stress are provided in the right column. Gray cubes represent tissue elements before deformation, and green deformed cubes with red planes represent tissue element when positive shear strains are applied. Yellow lines represent deformation angles.

Even though shear strain does not contribute to volumetric change due to the symmetric property of strain tensor, it provides additional information on three-dimensional growth during brain development. Similar to the results of JD, high and spatially non-homogeneous shear strains (Fig. 6) are observed during the first 6 months of life. Here, we showed the deformation angles exerted by shear strains between two originally perpendicular line elements (See Methods) to better visualize the effects of shear strains. For y-directional deformation exerting on yz-plane (*θ*_*xy*_), left and right hemispheres show opposite directions, gray matter tends to deform toward the anterior direction in the frontal lobe and toward the posterior direction in the occipital lobe (upper row). However, z-directional deformation exerting on xz-plane showed opposite directions in the anterior-posterior and inferior-superior directions; that is, gray matter at the inferior regions tends to grow downward while superior regions tend to grow upward (middle row). X-directional deformation exerting on xy-plane (*θ*_*zx*_) showed opposite signs in left-right directions, suggesting that gray matter tends to deform to the left direction in the left hemisphere and to the right direction in the right hemisphere (lower row). During month 6–12, all shear strains showed more subtle changes as observed in other parameters.

### Age effects on brain development profiles

**Figure 7:**
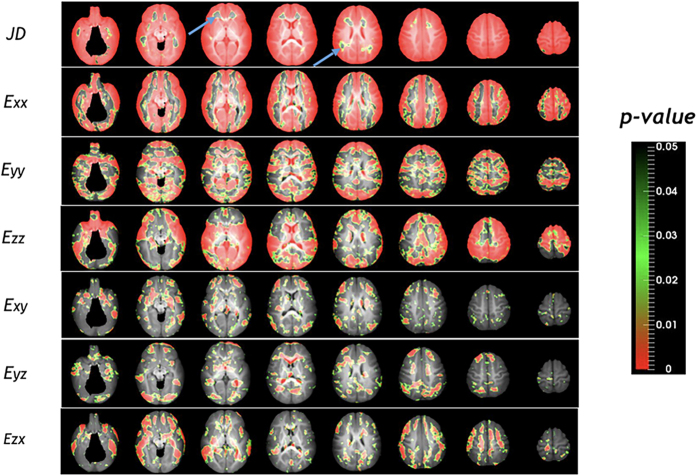
Age effects on volumetric and directional growth rates of the brain. Most gray matter regions show statistically significant age effects. These regions correspond to higher growth regions during the first 6 months. In most cortical regions, volumetric growth rate decreases with age. However, anterior and posterior regions of corona radiata don’t show significant age effect in volumetric growth rates (arrows). While directional elongations show significant age effect for most of the cortical regions, only limited regions show age effect in shear strains.

Effects of age on volumetric and directional growth rates estimated from a linear mixed effects model are shown in Fig. 7. Regions exhibiting statistically significant age effect are marked in red and green. With the exception of some areas at the anterior and posterior corona radiata, most of the gray matter regions showed age effect for JD. For directional elongations, direction-dependent age effects were observed in the gray matter, which is associated with high Lagrange strains for the first 6 months (Figs 2, 3, 4 and 5). The lateral ventricles, cuneus, and temporal poles showed statistically significant age effect for all directions. On the other hand, age effect on shear strains was observed only in limited regions of the brain. Finally, the slopes of the regression lines were always negative for brain regions exhibiting statistically significant age effects, indicating linearly decreasing growth rates with age.

## Discussion

The ability to reveal normal brain growth trajectories is of paramount importance not only to understand the nature of brain development but also to potentially improve the diagnosis of neurodevelopmental disorders. Although a wealth of imaging studies on early brain development has been conducted, most of the results to date have been derived from either a cross-sectional design or with a relatively long time interval between two scans, i.e., one year^3^,^16^, making it difficult to provide detailed characterization of brain growth trajectories. This study provides valuable normative insights into directional growth rates and their anisotropy using longitudinally acquired images with a relatively short time interval (3 months) between scans, capturing both non-linear and period-specific growth characteristics. To the best of our knowledge, our results offer the first directional growth information of the human brain during the first year of life by combining longitudinally acquired MR images and the finite strain theory of continuum mechanics.

As shown in the JD maps (Fig. 1), the brain volume growth patterns are time-varying and inhomogeneous during the first year of life. Compared with a previous longitudinal study^9^, similar volumetric growth patterns were observed: the slowest growing regions were sensory/motor regions and the fastest growing regions were insula and inferior temporal lobe. Slow and early postnatal growth of sensory/motor regions for the first year of life is possibly due to rapid maturation, which can be more precisely explained by the JD maps shown in Fig. 1. For the first 3 months, these regions show very high volumetric growth. However, after 3 months, the growth pace markedly decreased. Fastest growth rates at lingual gyri and inferior temporal lobes (higher-order visual processing)^21^ and fusiform gyri (involved with face recognition and color processing)^22^ can be explained by continuing local volumetric growth for all four periods of time. Corresponding to this inhomogeneous regional volume growth, directional elongations (Figs 2, 3, 4 and 5) exhibit region-dependent *anisotropic* growth patterns in most of the cortical areas, which could play an important role in forming the adult brain shape. The matured human brain is longer along the anterior-to-posterior direction and wider at the posterior part of the brain. This brain shape can be explained by anisotropic directional growth patterns in cortical regions during early infancy. During the first 6 months of life, active directional growths were observed (Figs 3, 4 and 5) and characterized by x-directional (left-to-right) stretch of the temporal lobes and interhemispheric fissure making posterior regions wider, y-directional stretch of frontal and occipital lobes making the brain longer in anterior-to-posterior direction, and z-directional (superior-to-inferior) stretch of superior temporal and occipital lobes enhancing the growth along the rostro-caudal axis as observed in mammalian neuroanatomical development^23^. Starting at 9 months old, markedly uniform directional growth patterns are observed (Fig. 2), which imply that the formation of adult brain shape is largely completed by 9 months of age. However, an alternative conclusion could be if one considers the C-shaped development of primate cerebral cortex and incorporates limbic structures into the temporal lobe. Future studies incorporating DTI could provide an additional means to investigate the relationship between growth direction and fiber orientation of the white matter.

Asymmetric growth patterns were also observed during early brain development (Fig. 1). This finding is interesting since it has been documented that cerebral asymmetry is present at birth (leftward asymmetry)^24^ while older children^25^,^26^ and adults show the opposite pattern (rightward asymmetry)^27^. Even though it is difficult to directly compare our findings with previous studies reporting volumetric asymmetry of the cerebrum^10^,^24^ due to differences in study design and scanning intervals, our results offer additional insights into the process from leftward asymmetry in infants to rightward asymmetry in older children and adults. Specifically, from month 0 to month 6, interhemispheric fissure regions of the right hemisphere showed active growth (Table 1). However, after month 6, asymmetric growth patterns became symmetrical with slow volumetric growth rates during this period. Nevertheless, more studies encompassing an age range from early infancy to early adulthood will be needed to provide more solid evidences on this issue.

The time course of synaptic overproduction and retraction using human brain autopsy specimens revealed that the peak of synaptic overproduction in the visual cortex occurs at about fourth postnatal month, followed by the auditory cortex, angular gyrus and Brocca’s area, and, finally, the peak overproduction of medial prefrontal cortex was observed around one year of age^28^,^29^,^30^. This synaptogenesis time course, to a large extent, is similar to the JD results observed in our study, demonstrating volume expansion during the first year of life. More importantly, results on normal strains offered additional insights into the underlying directionally dependent volume expansion. Visual cortex and auditory show active volumetric growth mainly driven by y- and x/z-directional elongations for the first 6 months, respectively. The vision processing and higher cognitive areas (lingual, fusiform, inferior temporal, superior parietal gyri) show higher volumetric growth driven by relatively higher z-directional growth profiles during months 6 to 12 (Figs 1 and 2). In contrast, superior frontal and middle frontal gyri that represent the dorso-lateral prefrontal cortex (DLPFC) showed a high volumetric growth during the first 6 months of life (*JD*^0–3^ > 1.78, *JD*^3–6^ > 1.39), which were associated with higher y- and z-directional stretches. Since it is well documented that DLPFC undergoes a prolonged period of maturation^18^, our findings may suggest that the initial increase in JD in DLPFC during the first 6 months of life may be more related to the overall brain volumetric increase but not necessarily functionally related. More studies covering a wider age range than that included in our study will be needed to further characterize the temporal behaviors of JD in DLPFC. After month 9, as shown in one-sample T-test results on JD maps (Fig. 1), the whole brain growth rate becomes uniform showing non-significant volumetric growth before synaptic pruning starts in early childhood. Additionally, the anterior and posterior horns of the lateral ventricles showed rapid and isotropic growth rates for the first 3 months (Fig. 2). This finding is consistent with a previous study reporting the temporal characteristics of ventricular shape changes during early infancy^16^. One should note that this study focuses on structural changes and can only infer to the synaptogenesis and pruning as reported by other studies.

Shear strains also provide important insights into brain growth. While normal strains (diagonal components) provide relative growth magnitudes along the main axes, we do not know, for example, whether a high z-directional growth of the lateral ventricles for the first three months is toward the superior direction or toward the inferior direction (Fig. 5). Referring to deformation angle *θ*_*yz*_ in that period (Fig. 6), it is apparent that downward growth (negative shear strain) is dominant in that period. Additionally, because shear stress is determined by shear strain and mechanical stiffness of the tissue, shear strains are potentially related to motility, proliferation, differentiation and survival of cells during brain development. Cells respond to mechanical signals in the form of externally applied forces and forces generated by cell–matrix and cell–cell contacts^31^. Here, we observed that shear strain is related to differential directional growth rates between gray matter and white matter. The white matter can be regarded as a fixed structure due to its lower growth rate in the first year of life while the gray matter exhibits an active growth during the same time period, leading to the observed shear deformations. Therefore, shear strain could play an important role in controlling the growth directions and local shape formation, interacting with directional elongations.

An increase of ICV up to two times of that at birth in the first year of life has been previously reported^3^. Regional brain volume increases have also been discerned using automated anatomical labeling-based volume analysis^9^ and showed marked volume increases (66.3–147.5%) in all brain regions. Consistent with previous reports, our study also reveals active volumetric and directional growth patterns during the first three months of life (Figs 1 and 2). However, one unexpected growth pattern was observed at the anterior regions of corona radiata showing non-significant volume change for the first year of life (Fig. 1), which is in marked contrast to the growth pattern of the majority of gray matter. This phenomenon can potentially be explained from both physical and biological points of view. Specifically, rapid growth of the lateral ventricles (280% increase) and gray matter (149% increase) have been reported; both markedly outpace the growth of white matter (11% increase)^3^ within the same time period. Structurally, white matter is located between the fast-growing lateral ventricles and gray matter. As a result, when the lateral ventricles push white matter outward, slow growing white matter will have to be stretched in response to the growth of the lateral ventricles. In addition, Bompard *et al*. reported a significant bilateral lengthening of the lateral ventricles and a significant increase of volume at the posterior portion of the ventricle during the first three months of life^1^. Together, these factors may attribute to growth patterns of the anterior corona radiata during the first three months of life showing significant local contraction in anterior-posterior direction (lower row of Fig. 2).

It is also of interest to compare infant brain growth patterns with those of a fetus. Fetal brains showed a higher volumetric growth in the frontal and temporal lobes with a major anterior-to-posterior directional stretch during 15–22 gestational weeks^32^. Greater volume increases in the parietal and occipital regions compared to the frontal lobes^33^ were observed during 20–28 gestational weeks. Postnatally, infant brains also exhibited similar growth patterns to those of fetal brains during the first 3 months. Subsequently, regionally specific growth directions are observed between 3–9 months, followed by more isotropic directional growth patterns around months 9–12 (Fig. 1). Interestingly, while the fetal brain showed volumetric contraction in the lateral ventricle in the second trimester, possibly due to increase in the thickness of the cortical mantle relative to the ventricles^32^, the infant brain shows the most active ventricular growth during the first year of life^1^,^3^.

In conclusion, biomechanical analysis using longitudinal MR imaging provides additional insights beyond commonly employed volumetric approaches. Furthermore, when *in vivo* mechanical tissue properties are available, driving forces for brain development can be further identified using the finite element model and deformation parameters investigated in this study.

## Methods

### MR Image acquisition

This study was approved by the Internal Review Board of The University of North Carolina at Chapel Hill and all research activities were carried out in accordance with approved guidelines. A total of 33 normal subjects (M/F = 16/17) were recruited and imaged using a Siemens 3T scanner (TIM Trio, Siemens Medical System, Erlangen, Germany) with a 32-channel phased array coil. Each subject was scanned at 5 time points: 2 weeks, and 3, 6, 9, and 12 months of age. Of the 33 subjects, 22 subjects were included in one of our previous studies^12^. Inclusion criteria were birth between gestational age of 35 and 42 weeks, appropriate weight for gestational age, and the absence of major pregnancy and delivery complications as defined in the exclusion criteria. Exclusion criteria included maternal pre-eclampsia, placental abruption, neonatal hypoxia, any neonatal illness requiring greater than a 1-day stay at a neonatal intensive care unit, mother with HIV, mother using illegal drugs/narcotics during pregnancy, and any chromosomal or major congenital abnormality. Informed written consent was obtained from the parents of all participants. Before imaging, subjects were fed, swaddled, and fitted with ear protection. All subjects were in a natural sleep state during the imaging session. A board-certified neuroradiologist reviewed all images to verify that there were no apparent abnormalities.

A 3D MP-RAGE sequence was used to acquire images with 144 sagittal slices at a resolution of 1 × 1 × 1 mm^3^. Imaging parameters were TR/TE/TI = 1820/4.38/1100 ms and flip angle = 7°. A Turbo spin echo T2-weighted sequence was employed to acquire 64 axial T2-weighted images at a resolution of 1.25 × 1.25 × 1.95 mm^3^ using TR/TE = 6750/20 ms, refocusing flip angle = 150° and turbo factor = 5. To mitigate the challenges of segmenting gray and white matter for images acquired at 6–8 months of age due to a poor gray/white matter contrast, diffusion tensor images consisting of 60 axial slices (2 mm in thickness) were obtained using an echo planar imaging sequence with imaging parameters: TR/TE = 7680/82 ms, matrix size = 128 × 96, 42 non-collinear diffusion gradients, and b = 1000 s/mm^2^. Seven non-diffusion-weighted reference scans were also acquired.

### Image registration and processing

**Figure 8:**
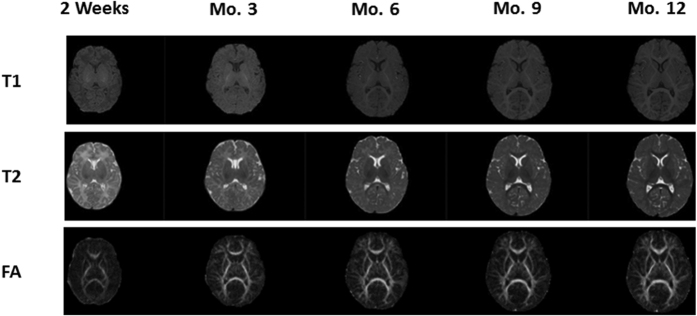
Representative longitudinal images of T1, T2 and FA for a single subject.

**Figure 9:**
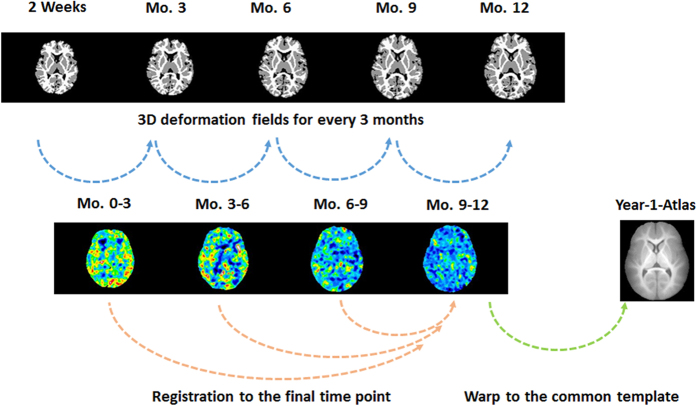
Estimation of growth rates (JD and Lagrange strain) of the brain for the first year of life. Using longitudinal segmentation/registration, 3D deformation fields of the 4 time periods (month 0–3, 3–6, 6–9 and 9–12) were derived. Volumetric and directional growth rates for every 3 months were estimated based on 3D deformation fields and finite strain theory. Parameters of growth rates were registered to the final time point of each subject and warped to the Year-1 infant Atlas for statistical analysis.

Longitudinally acquired multi-modality images (Fig. 8) from each subject were registered using the framework proposed by Wang *et al*.^12^,^34^. Preprocessing steps included skull stripping, N3-inhomogeneity correction, geometric distortion correction using field maps and non-linear registration. Subsequently, we conducted the within-subject longitudinal segmentation and registration consisting of the following two steps. (1) Single time-point tissue segmentation of MR images based on LINKS method^34^: the multi-source (T1, T2 and FA) images were used to iteratively estimate and refine tissue probability maps for GM, WM and CSF. As a learning-based approach, the segmentation framework consisted of two stages: training and testing stages. In the training stage, the classification forest was used to train a multi-class tissue classifier based on the training subjects with multiple modalities. The trained classifier provided the initial tissue probability maps for each training subject. The estimated tissue probability maps were further used as additional input images to train the next classifier, which combined the high-level multi-class context features from the estimated tissue probability maps with the appearance features from multi-modality images for refining tissue classification. By iteratively training the subsequent classifiers based on the updated tissue probability maps, a sequence of classifiers could be obtained. Similarly, in the testing stage, given a target subject, the learned classifiers were sequentially applied to iteratively refine the estimation of tissue probability maps by combining multimodality information with the previously-estimated tissue probability maps. Finally, to deal with the possible artifacts due to independent voxel-wise classification, we used patch-based sparse representation to impose an anatomical constraint^35^ into the segmentation. (2) Iterative 4D registration and segmentation^36^: after tissue segmentation of each time-point, a temporally consistent constraint term was incorporated to further guide the segmentation based on the fact that global brain structures of the same full-term infant are closely preserved at different developmental stages. The constraint kept the distance between the tissue boundaries of the serial images within a biologically reasonable range. Specially, for a given time-point, all other time-points images can be registered into the given time-point image space via 4D segmentation-based HAMMER. Therefore, the distances between the WM/GM (GM/CSF) boundaries in the given time-point image and other warped WM/GM (GM/CSF) boundaries should be within a biologically reasonable range. This temporally consistent constraint term was effective to guide the segmentation, especially for the time-point with the low tissue contrast. Similarly, with the improved segmentation results for each time-point, the accuracy of 4D segmentation-based HAMMER registration will be also improved. During the HAMMER registration, the later time-point with large volume was first resampled into the same size of each early time-point (or usually named linear registration) and then deformable registration was performed. In this way, each voxel in early time-points had a corresponding voxel in the later time-point. Then, parameter maps of JD and Lagrange strains were registered to the final time point of each individual (month 12) to keep longitudinal consistency of registration using deformation fields from month 3, 6, 9 to month 12, respectively (Fig. 9). At this stage, all within-subject longitudinal growth parameters for every 3 months were mapped onto his/her final images space. Finally, for group analysis, the deformation fields from subjects at month 12 to infant atlas at year 1^37^ were estimated using the same registration algorithm, which was applied to register all parameter maps onto the common space. Minimal smoothing (1 time edge-preserving smoothing) was applied at this step.

### Directional strain analysis using finite strain theory

The deformation gradient *F* is the fundamental measure of deformation in continuum mechanics. It is the second order tensor which maps line elements in the reference configuration to the one in the current configuration. In image registration, 3D deformation fields describe the voxel-wise displacements of each direction in the Cartesian coordinate system and through which deformation gradient tensor is defined by relating undeformed configuration (*X* coordinate) to deformed configuration (*x* coordinate):


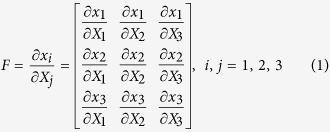


JD describes the volume change ratio between undeformed and deformed configurations:


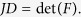


*JD* > 1.0 represents volumetric expansion whereas *JD* < 1.0 indicates volumetric contraction.

In continuum mechanics, one common choice for representing large strains between two different configurations is the Lagrange strain because it contains derivatives of the displacements with respect to the original configuration and can be conjugated with second Piola-Kirchhoff stress when the mechanical properties of material are known. Lagrange strain tensor is symmetric and describes directionally normal (diagonal components) and shear (off-diagonal components) strains:


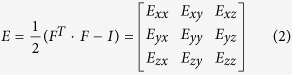


where *I* is a 3×3 identity matrix. When Lagrange strain *E* operates on a line element *dX,* it gives the changes in the squares of the undeformed (*dX*) and deformed length (*dx*):


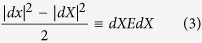


The eigenvalues of *E* are related to directional stretch (*λ*). When unit extension is defined as (|*dx*_*i*_|−|*dX*_*i*_|)/|*dX*_*i*_| = *λ*_*i*_ − 1, denoting the unit extension of *dX*_*i*_ by the normal strain, *e*_*i*_ in the direction *I*_*i*_, diagonal components of Lagrange strain are related to the normal strain; 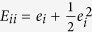. Positive values of Lagrange strain indicate stretch of line element after deformation and vice versa. From the relationship between *E*_*ii*_ and *λ*_*i*_, anisotropy of directional growth rates (ADG) can be described using the concept of fractional anisotropy.


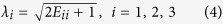



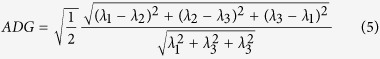


Off-diagonal components (shear strains) are symmetric and related to directional stretches and deformation angle, *ϕ*_12_:


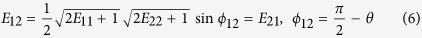


where, *θ* is the angle between two line elements that are organically perpendicular. Here, we need to note that Lagrange strain describes deformation based on the coordinate system of material particles before deformation.

### Statistical analysis

One-sample t-test was performed to determine if volumetric and directional growth rates are statistically significant during the four time periods (month 0–3, 3–6, 6–9 and 9–12). Here, null hypothesis is that JD = 1 or directional strain = 0 at each image voxel. To better discern brain regions revealing prominent changes in JD and strains (both normal and shear strains), 90 regions-of-interest (ROIs) using AAL template were defined^37^. Mean values of JD and strains of each ROI were computed and we defined the prominent growth region when the mean value of ROI was greater than 75^th^ percentile of all voxels in the brain including white matter and cerebrospinal fluid. Finally, the linear mixed effects model proposed by Laird and Ware^38^ was employed to determine age effects on the experimentally obtained longitudinal growth parameters using MATLAB R2013b (MathWorks, Natick, MA) software with the ‘*lme function*’. Specifically, voxel-wise analyses of the JD and 6 components of Lagrange strains (3 diagonal and 3 off-diagonal components) were performed considering ‘*Age*’ as a fixed effect and a random intercept for each individual. Since these parameters were estimated from different scanning intervals among subjects, all parameters were adjusted by dividing number of days between scans and multiplying 90 to represent changes within every 3 months^39^. Additionally, the age at the midpoint of each time period was considered as covariate^8^. Corrections for multiple comparison were accomplished whenever needed using False Discovery Rate^40^ for all of the above outlined statistical analyses and *p**-**values less than 0.05* were *considered* statistically significant.

## Additional Information

**How to cite this article**: Kim, J. C. *et al*. Biomechanical Analysis of Normal Brain Development during the First Year of Life Using Finite Strain Theory. *Sci. Rep.* **6**, 37666; doi: 10.1038/srep37666 (2016).

**Publisher's note:** Springer Nature remains neutral with regard to jurisdictional claims in published maps and institutional affiliations.

## Figures and Tables

**Table 1:** Fast growing regions where mean values of JD or direction strains were greater than 75^th^ percentile of the whole brain.

Period	JD	Exx	Eyy	Ezz
Mo. 0–3		Cingulum_Ant_L/R,	Cingulum_Post_R,	
		Cingulum_Mid_R,	Olfactory_L/R,	
	Cingulum_Post_R,	Cingulum_Post_R,	Lingual_L/R,	Occipital_Inf_R,
	Lingual_L/R,	Cuneus_R,	Occipital_Sup_L/R,	Fusiform_L/R,
	Occipital_Sup_R,	Precuneus_R,	Occipital_Mid_L/R,	Lingual_L/R,
	Occipital_Inf_R,	Frontal_Sup_Medial_R. Calcarine_R,	Occipital_Inf_L/R,	Heschl_L/R,
	Fusiform_R, Cuneus_R,	Frontal_Med_Orb_R,	Caudate_R,	Parietal_Sup_L,
	Precuneus_R,	Supp_Motor_Area_R,	Frontal_Sup_Medial_L/R, Cuneus_L/R,	Parietal_Inf_R,
	Frontal_Sup_Medial_R,	Rectus_R, Olfactory_R,	Fusiform_R,	Hippocampus_R,
	Calcarine_R,	Paracentral_Lobule_R,	Temporal_Pole_Sup_R	ParaHippocampal_R,
	ParaHippocampal_R,	Lingual_R,	Angular_R, Calcarine_L	Caudate_R,
	Hippocampus_R	SupraMarginal_R,		Temporal_Inf_R,
		ParaHippocampal_R,		Calcarine_R,
		Frontal_Inf_Tri_R		Temporal_Sup_R
Mo. 3–6	Cingulum_Ant_R,			Cingulum_Ant_R,
	Cingulum_Mid_R,	Cingulum_Ant_R,		Cingulum_Mid_L/R,
	Cingulum_Post_R,	Cingulum_Mid_R,	Cingulum_Ant_R,	Fusiform_L/R,
	Olfactory_R, Rectus_R,	Cingulum_Post_R,	Occipital_Sup_L/R,	Rectus_L/R,
	Fusiform_R,	Olfactory_R, Rectus_R,	Occipital_Mid_L/R,	Heschl_L,
	Occipital_Sup_L/R,	Fusiform_R, Insula_L,	Occipital_Inf_L/R,	Olfactory_R,
	Occipital_Mid_R,	Precuneus_R,	Olfactory_L/R,	Angular_R,
	Occipital_Inf_L,	Heschl_L/R, Lingual_R,	Cuneus_L/R,	Paracentral_Lobule_R,
	Angular_R, Heschl_L,	Frontal_Med_Orb_R,	Lingual_L/R,	Occipital_Sup_L/R,
	Insula_L, Cuneus_R,	Occipital_Inf_L/R,	Fusiform_R,	Occipital_Inf_R,
	Precuneus_R,	Temporal_Sup_R,	Angular_L/R,	Temporal_Inf_R,
	Frontal_Med_Orb_R	Temporal_Mid_L,	Parietal_Sup_R,	Temporal_Pole_Mid_L,
		Rolandic_Oper_R,	Calcarine_R,	Temporal_Pole_Mid_R Rolandic_Oper_R,
		SupraMarginal_L,	Temporal_Pole_Sup_R	Frontal_Sup_Orb_L/R,
		Amygdala_R,		Parietal_Inf_L/R,
		ParaHippocampal_R		Pallidum_R, Putamen_L,
				Insula_R
Mo. 6–9	Occipital_Sup_L,	Occipital_Inf_R,	Occipital_Mid_L,	Occipital_Inf_R,
	Occipital_Inf_R,	Fusiform_L,	Occipital_Sup_L,	Lingual_L/R,
	Lingual_L/R,	Temporal_Inf_R,	Angular_L/R,	Fusiform_L/R,
	Fusiform_L/R, Temporal_Inf_R,	Insula_L,	Cingulum_Post_L,	Temporal_Inf_R,
	Angular_L/R,	Parietal_Inf_R,	Olfactory_R, Lingual_L	Frontal_Sup_Orb_R,
	Parietal_Sup_R	ParaHippocampal_L		Parietal_Sup_R,
				Heschl_L,
				Rolandic_Oper_L
Mo. 9–12	Lingual_L/R,	Lingual_L/R,	Lingual_L/R,	Lingual_L/R,
	Fusiform_L/R,	Fusiform_L,	Fusiform_L/R,	Fusiform_L/R,
	Temporal_Inf_R,	Temporal_Inf_R,	Temporal_Pole_Mid_L/R, Temporal_Inf_R,	Temporal_Inf_R,
	Temporal_Pole_Mid_L. Parietal_Sup_R,	Cingulum_Post_R,	Parietal_Sup_R,	Parietal_Sup_L/R,
	Occipital_Inf_R,	SupraMarginal_L,	Rectus_L/R,	Occipital_Inf_R,
	Cingulum_Post_L,	Occipital_Inf_R,	Frontal_Sup_Orb_L/R,	Angular_L,
	Angular_L,	Heschl_L, Caudate_L, Frontal_Sup_Orb_L,	Frontal_Mid_Orb_L/R,	Paracentral_Lobule_L,
	SupraMarginal_L/R,	ParaHippocampal_R	Frontal_Med_Orb_R,	Cingulum_Post_L,
	Parietal_Sup_L		Rectus_R,	SupraMarginal_R,
			Fusiform_L/R	ParaHippocampal_R

**Table 2:** Slowly growing where mean values of JD or direction strains were less than 25^th^ percentile of the whole brain.

Period	JD	Exx	Eyy	Ezz
Mo. 0–3		Insula_R,		
		Frontal_Sup_R,	Cingulum_Ant_L,	
	Cingulum_Mid_L,	Frontal_Sup_Orb_L/R,	Cingulum_Ant_R,	Cingulum_Ant_L,
	Cingulum_Post_L,	Frontal_Mid_Orb_R,	Frontal_Inf_Tri_R,	Cingulum_Mid_L/R,
	Olfactory_L,	Heschl_R,	Temporal_Sup_R,	Frontal_Inf_Tri_R,
	Insula_R,	Hippocampus_L,	Amygdala_L,	Frontal_Med_Orb_L/R,
	Hippocampus_L,	Amygdala_L,		Olfactory_L/R,
	Amygdala_L	Putamen_L, Caudate_L,		Pallidum_R
		Pallidum_L		
Mo. 3–6		Amygdala_L,	Pallidum_L/R,	
	Pallidum_L,	Putamen_L,	Amygdala_L/R,	
	Thalamus_R,	Pallidum_L,	Paracentral_Lobule_L,	Hippocampus_L,
	Hippocampus_L,	Thalamus_L/R,	Precentral_R,	Thalamus_L/R
	Amygdala_L	Caudate_R	Postcentral_L/R,	
			Rolandic_Oper_L	
Mo. 6–9	Thalamus_L,	Pallidum_R,		Pallidum_R,
	Pallidum_R,	Thalamus_L,		Caudate_L,
	Calcarine_R,	Hippocampus_R,	Temporal_Pole_Sup_L/R,	Thalamus_R,
	Temporal_Pole_Sup_L/R,	Frontal_Med_Orb_L,	Rolandic_Oper_L,	Calcarine_R
	Temporal_Pole_Mid_R	Supp_Motor_Area_R,	Precentral_L	
		Paracentral_Lobule_R,		
		Cingulum_Ant_L,		
		Cingulum_Mid_L/R,		
		Olfactory_R		
Mo. 9–12	Hippocampus_L,			Hippocampus_L,
	Pallidum_L/R,	Pallidum_L,		Pallidum_R,
	Amygdala_L,	Cingulum_Ant_L,	Amygdala_R,	Amygdala_L,
	Calcarine_R,	Rectus_R, Olfactory_R,	Olfactory_L/R	Olfactory_L,
	Olfactory_L/R,	Frontal_Med_Orb_L,		Calcarine_R,
	Temporal_Pole_Sup_R, Frontal_Inf_Orb_R	Frontal_Sup_Medial_L,		Frontal_Inf_Orb_L/R
